# Language policy and planning: a discussion on the complexity of language matters and the role of computational methods

**DOI:** 10.1007/s43545-021-00206-6

**Published:** 2021-08-02

**Authors:** Marco Civico

**Affiliations:** Observatoire Économie Langues Formation, Faculté de Traduction Et Interprétation, Université de Genève, Geneva, Switzerland

**Keywords:** Language policy, Language economics, Computational methods, Simulation, Agent-based modelling, Public policy

## Abstract

In this paper I argue in favour of the adoption of an interdisciplinary approach based on computational methods for the development of language policies. As a consequence of large-scale phenomena such as globalization, economic and political integration and the progress in information and communication technologies, social systems have become increasingly interconnected. Language-related systems are no exception. Besides, language matters are never just language matters. Their causes and consequences are to be found in many seemingly unrelated fields. Therefore, we can no longer overlook the numerous variables involved in the unfolding of linguistic and sociolinguistic phenomena if we wish to develop effective language policy measures. A genuinely interdisciplinary approach is key to address language matters (as well as many other public policy matters). In this regard, the tools of complexity theory, such as computational methods based on computer simulations, have proved useful in other fields of public policy.

## Introduction

As a consequence of large-scale phenomena such as globalization, social integration, migrations, and progress in information and communication technology, the world has become a much more complex place than it used to be. Today, social systems, which I shall define *as collections of interacting components*, are much more interconnected, and actions within and outside these systems can (and often do) give rise to unexpected (or deliberately overlooked) reactions in terms of “what is reacting to what” and “with what intensity”. As a consequence, it is essential to understand in-depth such manifestations of complexity when analysing a wide range of activities, such as policy making. Indeed, policy interventions can produce consequences in apparently unrelated fields because of connections that have been overlooked or were simply unknown. Moreover, the issue that a specific intervention aims to address might have roots elsewhere, and therefore be resistant to policy intervention until these connections are clearly identified. Therefore, policy interventions in a complex environment are often effective only if they are designed with a degree of complexity that matches that of the issue they are addressing (Bar-Yam, 2015), lest societies collapse under a level of complexity that is no longer sustainable (Tainter, 1988). Clearly, this is true also for language policy issues. The complexity of human language and language-related social phenomena can be hardly denied. In this paper I provide a quick review of complexity theory, an approach specifically developed for the study of complex phenomena, and I put it in relation with language policy. My objective is to show that language policies have an intrinsically complex nature and should be studied from a complexity theory perspective. Besides, I want to discuss the potential contribution that computational methods can provide to the study of language-related matters.

Complexity theory is better described as a way of approaching the analysis of certain phenomena, a paradigm of study, rather than a theory *strictu *sensu. One should think of it more as a way of re-thinking things and phenomena, looking at them through a convex lens which enlarges the vision field. As we shall see in the following pages, complexity theory integrates a set of concepts and ideas derived from different disciplines and fields of research that give up a mechanistic view of the world in favour of a holistic approach, whereby the object of study is often characterized by a level of uncertainty. At the same time, this approach de-emphasizes linearity and predictability (Grobman, 2005). It places itself in direct opposition to the philosophical position of “reductionism”, which supports the idea that all processes and phenomena can be reduced to a collection of simpler basic parts. However, this does not amount to saying that complexity theory rules out the possibility of deducing larger macro-dynamics from individual micro-cases, quite the opposite. It simply states that a constant application of a strictly inductive logic risks being fallacious.

An in-depth discussion of complexity theory is far beyond the scope of this article. I will mention here only a few recurring traits of complex phenomena. Over the last decades, complexity has been defined in a number of different ways, each definition stressing one aspect or another. A complex system has a *large number* of *heterogeneous*, independent and *interacting components*, able to evolve along *multiple pathways*. Its development is very *sensitive to initial conditions* or to small perturbations, and it is analytically described through *non-linear* differential equations (Whitesides and Ismagilov, 1999). On top of being inherently complicated, complex populations are rarely deterministic, and predisposed to *unexpected outcomes* (Foote, 2007). The major problems for those who get to deal with complex systems are *unintended consequences* and the difficulty of making sense of a situation (Sargut and McGrath, 2011). All of the emphasized words in the previous sentences are highly recurrent in theoretical and applied texts dealing with complexity theory. At times, each of these aspects have been highlighted as the key characteristic of a complex system.

In the coming pages, I first provide a definition of language policy and why language matters are usually addressed through policy. Then, I go on to discuss complexity. However, instead of providing an in-depth description of the numerous aspects of complex phenomena, I will review some of them by referring directly to language-related issues. Finally, I discuss the role that computational methods can play in language policy making.

## Language policy and complexity

Public policy can be defined as “*an intentional course of action followed by a government institution or official for resolving an issue of public concern*. Such a course of action must be manifested in laws, public statements, official regulations, or widely accepted and publicly visible patterns of behaviour” (Cochran et al. 2009, pp. 1–2, emphasis in original). The expression “of public concern” reveals the collective nature of the issue at stake. Language is the natural means of communication, which is an inevitable part of society. As a consequence, language issues and all their social, political, and economic implications deserve the attention of public policy practitioners and scholars. The role played by policy analysis in the field of language policy has been evident to scholars (in particular to sociolinguists) since the 1970s (Jernudd, 1971; Rubin 1971; Thorburn 1971). Nevertheless, it received greater attention only starting from the 1990s, when a number of scholars from political science and economics started to apply policy analysis models to language policy (Grin and Vaillancourt 1999; Grin and Gazzola 2010; Gazzola 2014).

But what is *language policy* exactly? This question is rather simple and might pop up spontaneously in the mind of those who are not technically involved in it. However, language policy is something that affects everyone, passively and actively. It is fundamental to understand that almost anything involving communication and language use is the result (at least in part) of different combinations of language policy measures, from the choice of providing certain services in a given language to the drafting of school curricula. Sometimes, even the linguistic identity that an individual assigns to herself and her community might be influenced through policy measures. Besides, language policies have repercussions on society which might affect people’s life so profoundly that it is often very hard to isolate them. Suffice it to say that numerous researchers in the social sciences (and not only) often find themselves facing language issues in their daily work and have a hard time managing them. The non-negligible impact of language policies on people calls for sustained research efforts completely focusing on them. Furthermore, it is impossible to deny the importance of language policy and its very existence, as there is no reality involving communication between humans with “no language policy” — as a matter of fact, the simple fact of declining to take decisions concerning language issue *is* a form of language policy (which is being, anyway, communicated in a certain language).

However, providing an answer to the questions concerning the very existence of this field of research (such as: Why is it necessary? What are the objectives? What are the material results of this research?) has proved, over the last few decades, somewhat difficult. The cause of this is the lack of a generally accepted comprehensive theory of language policy. However, this is not the consequence of superficial research, but rather the opposite. Language policies are so entrenched in everyday life that they are acknowledged and practiced in all societal domains. As Ricento puts it,

“[w]hile [language policy (LP)] as an organized field of study is a relatively recent development, the themes explored today in LP research have been treated in a wide range of scholarly disciplines in the social sciences and humanities over the years.” (Ricento, 2006, p. 19)

### Are language issues complex?

I will now reconsider language policy and some notable language-related issues from a complex perspective in order to show that language issues display the typical traits of complexity and, therefore, that they “qualify” to be considered and treated as complex issues. The direct consequence of the acknowledgement that language issues are complex implies that language policies have to be drafted in a way that takes the principles of complexity theory into account. This does not mean “changing language policies”, in that a policy is not something that exists autonomously, in its own right. A policy is an answer to a specific problem, without which it has neither reason nor legitimacy to exist. Therefore, a policy answer should reflect the characteristics of the object being treated. If we recognize that language issues are complex, the policy maker needs to adopt a complex approach to draft an answer. I provide examples illustrating different language issues. My objective is to prove that language issues are complex in their nature and therefore should be addressed by means of complexity theory.

#### Non-linearity and feedback loops

By non-linearity, specialists of complexity often mean that a system displays a fundamental disproportionality between cause and effect (Homer-Dixon, 2010). In a simple (linear) system, a small disturbance implies a small change, while a big disturbance leads to a big change. On the contrary, complex systems do not necessarily exhibit such proportionality. Small changes could imply dramatic effects and big ones could have only marginal implications. Scheffer et al. (2001) propose an interesting example from ecology to explain non-linear responses to external stimuli. They note how an ecosystem (in particular, they focus on the eutrophication process of shallow lakes, i.e., the process by which shallow lakes become overly enriched with nutrients, therefore inducing excessive growth of algae) may appear to remain unchanged or only slightly changed up to a certain critical level of human intervention (which they name “stress”), beyond which follows a catastrophic transition to a new state. However, a switch back to the previous state (in the case studied by Scheffer et al., 2001) requires going back much more than the simple restoration of the stress level preceding the collapse. Another good example is desertification. Increasing grazing intensity can incrementally destroy vegetation, but once desertification occurs, it is not enough to reduce (or even remove) grazers in order to stop it and restore the previous level of vegetation.

A language-related example could be the process of acquiring new vocabulary. Indeed, vocabulary learning begins at a very slow rate, which then increases and eventually slows down again (Larsen-Freeman and Cameron, 2008). If we were to plot this pattern on a graph with time on the x-axis and vocabulary size on the y-axis, we would not draw a line but an S-shaped curve. At the beginning it is reasonable to find it difficult to acquire new words, especially if they are substantially different from the vocabulary of one’s native language. One might find it hard to memorize new and possibly unfamiliar sounds and orthography, which make the acquisition process very slow. However, as the process goes on, one becomes more and more familiar with pronunciation and spelling and might even start making connections between words sharing the same root or having the same prefix or suffix. Besides, it is very likely that a person interested in learning vocabulary in a new language is exposed to that same vocabulary in other ways, both actively and passively, for example hearing it at the grocery store, reading it on billboards, and so on. When talking specifically of young children in the process of learning their native language, MacWhinney (1998) factors in growing cognitive capacity as an additional interaction variable. All this clearly accelerates the acquisition process. However, as one reaches a wide vocabulary, the process slows down again and approaches a horizontal asymptote. This can happen for a number of reasons. For example, it becomes less likely that one hears or reads less frequently used words; or, being able to count on a wide vocabulary and sufficient fluency to make oneself understood, one might feel less and less in need of looking up a specific term on the dictionary, eventually missing out on least common synonyms.

Several instances of non-linear patterns have been detected by scholars in language policy and economics of multilingualism. A well-known example is that of the threshold in the process of language shift, i.e., the process whereby a speech community traditionally speaking language B, gradually replaces it with language A (Grin, 1992). A threshold is a stage in this process where “it is too late” to go back, a point where language B will now inevitably give way to language A. Ambrose and Williams (1981) discuss the case of Welsh. Distinguishing between Welsh-speakers (including bilinguals) and Welsh monoglots, they argue that there exists a “language loss line” (slightly below 50% of monoglots, according to their empirical findings) under which the entire Welsh-speaking population starts to drop and eventually disappears. Grin (1992) further explored this intuition from a theoretical perspective, noting that there is no single threshold point. Rather, several (or better, an infinite number of) “points of no-return” exist, depending on the interaction of demographic and linguistic variables, such as the distributions of speakers across languages (in its turn affected by migration flows as well as birth and death rates) and the attitudes of people towards these languages (depending, among other things, on the availability of opportunities to use a specific language). Language survival can be attained through policy intervention. The function linking these variables tends to be non-linear. Besides, a small variation in the initial condition leads to drastic changes in the stable equilibrium eventually attained. Therefore, any action drafted by policy makers should take this non-linearity into consideration. In the same study, the author identifies a feedback loop characterizing the level of language survival (defined by a variable called “language vitality”). In particular, the latter is quite clearly influenced by intergenerational transmission as well as individual loss and acquisition because they determine the percentage of speakers of each language. At the same time these two variables are functions of language vitality. A decreasing level of language vitality can induce a decline in the level of intergenerational transmission, as well as in the level of acquisition, which eventually cannot make up for the loss of speakers over time (of course, the opposite is also true, in that an increase in language vitality increases the interest to transmit of acquire the language). Grin (1992) notes, however, that language vitality does not necessarily feed on itself.

#### Non-gaussianity

A non-Gaussian distribution is characterized by a higher-than-normal likelihood of extreme events, which can bring about unexpectedly big effects. The fact that extreme and unexpected events can have important consequences on language issues is easy to see. In the previous pages it was briefly mentioned that migration flows play a role in the definition of the linguistic landscape of a region. This is true both in the long-term and in the short term. In the long-term, one can think of colonization processes that made four European languages (English, French, Spanish and Portuguese) the major languages of the contemporary Americas, virtually annihilating indigenous languages.^1^ Concerning the short term, one can think of the emergence of non-indigenous communities across Europe over the last few decades (either non-native to the country of settlement, such as the Romanian community in Italy, or to Europe in general, such as Latin American or African communities). This clearly has implications from a number of perspectives. One can think of the EU directive that ensures the right of suspected or accused persons to interpretation and translation in a language that they understand during criminal proceedings.^2^ As a consequence, member states have an obligation to provide interpreters and translators to people speaking a language different from the local one(s). Complying with this principle is straightforward. Locating and anticipating needs is relatively easy, and so is preparing and eventually providing competent professional to meet the requested services. However, this is only simple as long as the language landscape remains constant or changes in a “predictable” way. Nevertheless, such a system may collapse very easily under the pressure caused by an (apparently) unlikely and unforeseeable event. Current migration flows seem to confirm this. Because of unexpected events (such terrorism and war in the Middle East and a long civil war in Libya), migration flows towards Europe have dramatically increased during the last few years, boosting the presence of non-indigenous people on European soil, from all sorts of different cultural and linguistic backgrounds. Such an unpredicted shock can easily undermine the functioning of the administration (not to mention socio-economic repercussions). A sudden increase in the volume and diversity of migration is not easy to cope with when it comes to granting T&I services, among other things. The receiving country may not be prepared in terms of staff to deal with incoming people. As a consequence, policy interventions in this domain should aim at boosting systemic resilience by making it flexible and able to quickly adapt to external shocks.

In the case of complex phenomena, extreme events occur more frequently than a Gaussian distribution would predict and, most importantly, they carry more weight than one could expect. Besides, if we concentrate on the average of the observed values, we might be missing an important part of the story. In complex phenomena, seemingly unlikely events are not that unlikely and can have dramatic repercussions. In general, one might be tempted to use historical observations to make predictions, assuming the existence of predetermined patterns which will eventually repeat themselves over time, in a cyclical fashion. This is not the case for complex systems, where outliers often have significant consequences. In relation to complex systems, scholars have sometimes spoken of “black swans”^3^ to define those occurrences that are believed not to be possible until they actually occur. Besides, it can be argued that these events are the only ones that can seriously affect a system and have a long-term impact (such as sudden shocks in the financial markets).

I shall devote a few words to clarifying the difference between non-linearity and non-Gaussianity, as they can be easily mistaken. Both ideas focus on the fact that apparently minor issues can have important consequences. However, non-linearity is about the magnitude of the impact of small events, regardless of their likelihood or frequency. Non-Gaussianity, conversely, only reminds us that, in complex systems, one cannot rule out extreme events, if only because they are often the ones that imply the most significant impacts.

#### Spontaneous order and lack of central control

Spontaneous order and lack of central control are possibly the easiest complex characteristic to spot, concerning both language itself and language use. A language evolves over time, developing a rich vocabulary and a complex syntax with every speaker (often unconsciously) contributing to it (Cantor and Cox, 2009, p. XI). Speakers reciprocally give up a part of their linguistic liberty (intended here as the ability to pronounce different sounds) to “meet halfway”. They define common words and rules in order to be able to understand each other (Adelstein, 1996). However, these rules are created, followed and broken continuously. As was observed above, a living language is never in equilibrium. Rather, it fluctuates around an “equilibrium region”, determined by a spontaneous tendency to maintain mutual intelligibility among speakers, and by individual use, whose peculiarity are often defined at a decentralized level. Besides, it should be noted that languages are often resistant to central control, i.e., the attempts of language scholars to regularize speech patterns (Cantor and Cox, 2009, p. XII). If, say, a language regulator prescribed the use of a specific word or grammar rule, it is not obvious that speakers would respond positively to the imposition. It is the case, for example, of adjective agreement in many Romance languages, such as French. While the Académie française, the central institution that deals with matters pertaining the French language, prescribes the use of the masculine agreement when an adjective refers to a number of nouns with different genders, some users display a preference for a more inclusive language using, among other things, the so-called “accord de proximité” (proximity agreement). Such type of agreement provides that adjectives should agree in gender with the closest noun.^4^ Therefore, it is evident that a random element coexist with a spontaneous order and a weak form of central control.

#### Emergence and hierarchical organization

Emergence is probably one of the (if not the single) most important characteristic displayed by complex systems and would probably deserve a whole discussion to itself. Here, however, I only offer a general introduction. A system is characterized by emergence if it exhibits novel properties that cannot be traced back to its components (Homer-Dixon, 2010). Some scholars call these properties “emergent properties” (Bunge, 2003; Elder-Vass, 2008). The adjective “emergent” refers to the fact that such properties are not present at the individual level, but only “emerge” as we move on to consider higher levels of aggregation. To understand this idea, one could think of utterances as sets of words. Words have their own properties (such as meaning and syntactic function) and, put together, they can form sentences. However, a sentence is more than the simple sum (or succession) of the words that it contains. It has its own meaning that emerges only when its components are put together and is also dependent on extra-verbal contextual elements.^5^

A good example from the natural sciences is the saltiness of sodium chloride (i.e., table salt), which is not attributable neither to chloride nor to sodium individually. Saltiness *emerges* as a consequence of a (1 to 1) combination of the two elements. Elder-Vass (2008) goes on to stress that an emergent property is not only one that is not possessed by any of the parts individually, but also one that would not be possessed by the compounded entity if there were no structuring set of relations between the individual parts (and it is therefore not due to the mere co-presence of these elements). This reasoning echoes what Nobel laureate Herbert Simon argued much earlier:

“Roughly, by a complex system I mean one made up of a large number of parts that interact in a non-simple way. In such systems, the whole is more than the sum of the parts, not in an ultimate, metaphysical sense, but in the important pragmatic sense that, given the properties of the parts and the laws of their interaction, it is not a trivial matter to infer the properties of the whole.” (Simon, 1962, p. 468)

One could conclude that all other characteristics of complex systems are indeed emergent properties. As a matter of fact, that is far from being incorrect. Spontaneous order and self-organisation, discussed in the previous subsections, are indeed emergent properties. They emerge only as a consequence of the existing interactions between parts and they are not inherent to any of them. Talking specifically about spontaneous order, Hayek defined it as “orderly structures which are the product of the action of many men but are not the result of human design” (Hayek, 2013, p. 36). Market dynamics leading to equilibria (in the absence of a central coordinating body) are quite an eloquent example of emergent (orderly) behaviour (Petsoulas, 2001).

To explain emergence within language issues, I consider the issue of clashing interests at different levels, which underlines the importance of taking also the meso-level into consideration. In general, recognizing the complex nature of language matters and adopting a tripartite perspective becomes crucial for policy makers. Let us look at another practical example, building on a discussion by Grin (2015) on the use of different languages in a higher education context:

- at the micro-level, a researcher walking in a (at least moderately) culturally diverse university will immediately notice that individual students have different backgrounds, different language profiles, and use all sorts of different communication strategies (speaking their own language, speaking the interlocutor’s language, code-switching, code-mixing, intercomprehension, and so on) and that these strategies are adopted by users with no external restriction;
- at the meso-level, a researcher will notice that universities make choices about the use of one or another languages for different purposes (e.g.: choice of languages taught as subjects; choice of language(s) of instruction, including exams and, possibly, educational materials; choice of language(s) for internal administrative purposes; choice of language(s) for external communication) and that these choices do not necessarily corresponds to micro-level strategies in terms of diversity;
- at the macro-level, an LPP researcher’s interest will typically concern the general choices made by the authorities (assuming we are dealing with a publicly funded education system) regarding the language(s) of instruction in universities (as well as in other educational contexts).
  We shall also note that interests may actually coincide between the micro and the macro-levels and that therefore we would be missing a big side of the story if we ignored meso-level entities. Let us consider country X, where X is the official language, though Y is also spoken by a newly formed community, whose members are not always fluent in X. Besides, country X has significant trade relations with country Z. Let us also consider the information written on the packaging of goods for sale in country X’s supermarkets. Such information includes ingredients, conservation methods, origin, etc. A breakdown of the interests at stake in this scenario based on the three levels of perspective will be as follows:
- MICRO: individual A, speaking exclusively language X, doing shopping in the local supermarket. She is clearly interested in understanding what is written on the packaging and, therefore, she wants and, as a national of country X, *expects* information to be provided in a language that she speaks, i.e., language X. In another aisle of the same supermarket, individual B, belonging to the immigrant community and speaking language Y, *would like* to have information in language Y, but is willing to struggle with language X.
- MESO: the CEO of a company based in country X producing goods to be sold in supermarkets. Incidentally, the CEO is also part of the immigrant community, her native language is Y, but she is also fluent in X. As she acts on behalf of a private institution, it is reasonable to assume that her sole interest is to generate profit for the company and, therefore, she would want to limit as much as possible packaging costs, which include printing information. Initially, she would avoid completely adding information in another language, but she fears that the company might lose clients. In country Z, another CEO is facing a similar situation. She would like to market the company’s product in country X, but this would imply translation costs (towards X? Y? Both X and Y?), packaging reconfiguration and, therefore, an increase in production costs.
- MACRO: the president of country X’s consumer protection authority. She was appointed by the newly elected government, a notable a supporter of minority rights. Her main interest is, obviously, that consumers are protected and, therefore, constantly aware of their consumption choices. She works to push the parliament of country X to pass a law that obliges companies selling products in country X to provide information in language X on the packaging, whether the company is local or based abroad. At the same time, she is working on another proposal that would introduce an obligation to add information in a non-official language spoken as a unique language by more than a certain amount of tax-payers. However, she did not put it in the law about language X because she did not want to provoke a negative reaction from the opposition.

A number of considerations can be made on the basis of this example. We note that interests at the micro and macro-levels are somewhat converging, in that in both cases the optimal solution would be to have information the language(s) spoken by the residents of country X. If one skipped the meso perspective, one would be tempted to believe that this is where the story ends, but we would be overlooking a whole other set of interests. At the meso-level we find entities such as corporations who have completely different objectives and might even consider the request for multilingual information a nuisance. It is evident that the fact that the micro and macro interests converge is not enough and that intervention at the meso-level is needed. One might argue that the companies of the example are private actors and that their behaviour cannot be determined through policy making (as far as language use is concerned). This is only partially the case. True, these private institutions have freedom to make decisions about language use as far as internal processes are concerned. Nevertheless, the government can (and often must) intervene to regulate the relationships between these companies and the people, including the use of language. Finally, we should note that the company’s profit is of interest for every individual in the company, our meso-unit, whether they are nationals, foreigners, speakers of language X or Y. Indeed, the company’s success (or failure) has substantial repercussions on its workers’ conditions. Therefore, we can note how, as mentioned, a meso-level characteristic (in this case, the interest for corporate profit and the subsequent strategies) come into existence only when the meso-level aggregation takes place.

## The role of simulation in social science research

So far, I have shown that language issues display, among other things, non-linear behaviours, spontaneous order and emergence. Besides, extreme and unlikely events can have dramatic repercussions. Consequently, policies dealing with language issues should be drafted adopting a specific complexity approach. This is particularly true considering that simulations are a good substitute for real-life experiments when these are potentially expensive and burdensome. The literature on complexity theory offers a good number of examples of applications of complexity theory to public policy matters. However, complexity theory is only seldom applied to language policies. Therefore, as of today there is no such thing as a complex framework to implement language policies, able to address language challenges in a flexible and adaptive way, taking all non-trivial aspects into consideration. The general idea is that, due to the non-negligible presence of randomness, language policies (and, in general, policies addressing situations where the future in unpredictable) call for a complex approach. Therefore, traditional quantitative and qualitative methods need to be complemented by other research methods, such as computer-based simulation. In particular, agent-based models have the great advantage of relating the heterogeneous micro-behaviours of agents with different information, decision rules, and situations to the macro-behaviour of the overall system (Lempert, 2002).

To address the difficulties posed by complex systems, researchers often resort to large-scale controlled studies for the purpose of spelling out individual causal links. When it comes to the study of social phenomena, however, such investigations are often not possible, for a number of practical and ethical reasons. Besides, “controlling” a group of people in their social interactions is not the same as controlling, for example, the way they are treated with a specific drug, not to mention controlling the behaviour of particles or molecules. Humans and their behavioural patterns can vary in virtually countless ways and very similar conditions can sometimes lead to radically different results. It is very hard to isolate social systems from the influence of the greater network in which they exist. For example, it is not sufficient to study the behaviour of the students of a given school without considering the city in which the school is located. This makes it virtually impossible to rule out the impact of external causes on the dynamics under examination. Besides, this becomes all the more critical as global interconnectedness and interdependence increase. In short, a purely in vitro study is usually not possible in social science research. Sometimes, researchers get around this problem by resorting to theoretical modelling, which is highly mathematics-based and, thanks to its inherent formality, helps spelling out causal links with a high degree of conceptual consistency. Theoretical modelling is, in a certain sense, a way of “controlling” the experiment, in that the modeller can make assumptions about properties and behaviours. However, this still does not solve the problem of mutual influence with other systems, nor does it account for the fact that individual human beings are extremely heterogeneous in their properties. Analytical models often need to put aside such heterogeneity for the sake of mathematical tractability. This is where computational social science, the field of social science that resorts to computational methods, comes into play. Among computational methods, agent-based modelling, a type of computer simulation method, is a particularly important ally of social scientists. Agent-based models use algorithm to simulate the behaviour and interactions of micro-level agents with a view to replicating some macro-level dynamics under study.

The general idea behind computer simulations for the social sciences is relatively straightforward. Given that it is often impractical to realize large-scale controlled studies, social scientists can resort to computer simulations to recreate an *in-silico* version of the context of interest and simulate the dynamics considered. The environments and the agents are usually informed through real-life observation, so as to make the model representative of reality. If the model is conceptually coherent and its behaviour is validated by actually observed trends, one usually goes on to study the dynamics of the system under different conditions. In so doing, one can evaluate how and to what extent different variables impact the overall system.

Applications of agent-based modelling have been increasing at an accelerating pace and concern a wide range of fields. Simply looking at a few recent publications in which agent-based modelling was the main methodology, we find applications as varied as:

1. a simulation of firms' decision-making processes with a view to detecting the relation between the heterogeneity of firm sizes and innovation stemming from collaborative behaviour (Hwang, 2020);
2. a simulation model investigating how different policy interventions contribute to the use of electric vehicles and the use of renewable energy sources to recharge them (van der Kam et al., 2019);
3. a simulation of people's meat consumption in Britain and the different impacts of price changes, animal welfare campaigns and health campaigns on people's propensity to consume meat (Scalco et al., 2019).

It should be clear, then, that computation-based methods are an important ally for social scientists. Obviously, they are not meant to replace other methods, such as equation-based and statistical models, but rather to complement them, in order to put the massive progress in information technology at the service of research. As a matter of fact, simulation models rely heavily on other methodologies both in the development part and in the analysis of the results. As an example of such practice, Carrella et al. (2020) discuss the application of linear regularized regression to find the optimal calibration of the model parameters to match the data. Starting from the fact the regression is a well-understood and commonly used method, the authors leverage this knowledge and apply it to the delicate task of parameter estimation. In short, they propose following these four steps:

1. repeatedly run the model with a random vector of K parameters at every simulation;
2. collect M summary statistics for each simulation;
3. train K different regressions using each parameter as a dependent variable and the collected summary statistics as independent variables;
4. finally input the actually observed statistics in the K regressions in order to find the "real" parameters that generated them.

For another example, ten Broeke et al. (2016) review in great detail the pros and cons of various methodologies to perform sensitivity analysis on agent-based models in terms of different aims. The three methodologies analyzed are:

1. regression-based methods, which decompose the variance of the ABM outcomes by regressing them against the input parameters;
2. the OFAT (one-factor-at-a-time) sensitivity analysis, which looks at the variation in the output when one parameter changes, while all other parameters are kept fixed;
3. the so-called Sobol method, which decomposes the overall variation in the model by attributing fractions of it to individual parameters.

The aims taken into consideration by the authors are the following:

1. to find how patterns and emergent properties are generated within the model;
2. to examine the robustness of emergent properties;
3. to quantify the variability in the outcomes resulting from model parameters.

It is clear, then, that agent-based models, as well as simulation models in general, do not represent an alternative to other more traditional research methods. Quite to the contrary, their potential is fully exploited when they are used in combination with other methods.

### The role of simulation in language policy

In light of the considerations made throughout this paper, it should be clear that language-related phenomena unfold in a complex environment. Indeed, as was seen, language issues are never *just* language issues, which is why they should always be studied from an interdisciplinary perspective. It is enough to look at some recent publications in the field of language policy and language economics to realize how language matters are strictly connected to numerous seemingly unrelated areas.^6^ For example:

1. Golesorkhi et al. (2019) examine the relationship between language use and financial performance of microfinance banks;
2. Civico (2019) discusses the use of language policy to serve socio-political objectives throughout the twentieth and twenty-first centuries in China;
3. Kang (2020) analyses the changes in North Korea’s language policy and attitudes towards the English language following the rise to power of Kim Jong-un.

To give an idea of the numerous ways in which language matters can be articulated, Grin et al. (2018) address 72 questions concerning languages organized in six different sections. The topics included range from language policy analysis to linguistic diversity and language education. The collection of questions was addressed by teams of people having different disciplinary backgrounds, ranging from economics, mathematics and philosophy, to education, sociolinguistics and law. This comprehensive approach stems from the realization that language issues are all interrelated and exist in a greater system. Issues such as language teaching, the provision of language services, the protection of minority languages and the official adoption of a language all influence and are influenced by each other. In light of all this, a complex perspective on language matters becomes crucial if one hopes to gain more complete and deeper insights. Ideally, this would be achieved by setting up large-scale studies involving numerous people with different disciplinary backgrounds. However, this is not always possible, in that it calls not only for a conjoint and coordinated effort, but also for substantial financial support. This is where computational modelling comes in particularly handy. Thanks to their flexibility and capacity to integrate knowledge from various fields, simulation models allow us to gain insights into dynamics that would otherwise be unobservable. Considering the converging evidence about the complexity of language matters adduced throughout this dissertation, it seems reasonable to conclude that, as is the case for many other fields, language policy can benefit from the application of a complex approach. An optimal implementation and evaluation of language policies (as well as policies in general) requires a large amount of data and, ideally, direct observation of the impact. However, this is not often possible, and in many cases, it is not advisable to implement a measure just for the sake of observing its effect. Agent-based modelling offers a natural solution to such problems. It can help language policy makers in at least three different ways:

1. by simulating existing phenomena to gain insights about the matter under study (such as the development of different communication strategies);
2. by providing an assessment of the potential impact of different measures (for example, investigating how an increase in the average level of fluency in a minority language affects the number of speakers over time);
3. by simulating the changes in the system caused by exogenous shocks (such as the impact of a sudden wave of immigration on the linguistic landscape).

All these objectives can be achieved by policy makers by drawing from and building on the already vast amount of qualitative research on language matters. Indeed, agent-based modelling is a natural extension of qualitative studies. Besides, as I will discuss in the next section, ABMs do not need to rely exclusively on social theories to provide agents with realistic behavioural rules. Agent-based modelling is very flexible and can be easily combined with other more qualitative-oriented methodologies.

Among the virtues of agent-based modelling, I highlighted aspects such as flexibility, adaptability, effective visualization, ease of programming and immediate usability by both experts and non-experts. However, I would like to mention another strength of ABMs, one that may speak especially to policy makers, i.e., their ability to capture potential unintended consequences of policy measures. Unexpected or unintended effects of policies are rather common and discussed at length in the relevant literature. For example, Bernauer and Knill (2009) investigate the case of a German packaging waste policy that turned out to be ineffective soon after its implementation and that proved very hard to dismantle. Unintended consequences usually result from a combination of complexity and lack of information that limits policy makers' understanding of the policy (Lindkvist et al., 2020). They can be frustrating, confusing, and time-wasting. Most importantly, unintended consequences can be costly.

The issue of unintended consequences is crucial for policy makers, who are often reluctant to put in place costly large-scale policy measures on the basis of theory-based models that can only be verified after implementation. However, ABMs can provide a risk-free environment in which policy makers can experiment with different measures. Indeed, if developed with sufficient attention to social and behavioural mechanisms, an ABM can highlight some unexpected or unintended dynamics thanks to its integrated multi-scale environment. In practice, this would amount to saving a non-negligible amount of money that would have otherwise been invested either in testing practically the theory-based policies or in developing and implementing measures aimed at fixing or even reverting the unintended effects of the policy. Therefore, the integration of agent-based modelling in the current policy making process can result in non-negligible savings, better resource allocation and generally improved governance.

### Potential applications

#### Agent-based modelling and fuzzy logic

In an attempt to increase the level of realism of simulation models, some authors have suggested combining agent-based modelling with fuzzy logic as a further extension to the use of natural language data to inform agents. Fuzzy sets are sets in which elements have a "certain degree" of membership. Differently from Boolean logic, in which membership may only take one of two values, i.e., 0 (not a member) or 1 (member), in a fuzzy framework, an element can belong to a set with a varying degree of intensity. In short, each member of the set takes on a "grade of membership" that ranges from 0 (not a member) to 1 (full member). All members taking on values in between are "partial" members. Fuzzy logic is able to capture the vagueness and uncertainty which often guide human behaviour. In the perspective of using textual data to define the behaviour of artificial agents, being able to discern the intensity of people's attitude with respect to specific facts can be crucial. Besides, also the extrapolation of agents' properties from text data can greatly benefit from the use of an approach that can deal with uncertainty. After all, humans constantly function with a certain degree of uncertainty. Izquierdo et al. (2015) propose the following example. Consider the sentence "a tall, blonde, middle-aged guy with long hair and casually dressed is waiting for you at *(sic)* the lobby". While a human can more or less easily figure out who the concerned person is in a group of people (or at least narrow down the selection to a number of elements), implementing an equally effective artificial agent can be extremely challenging. The reason is that concepts such as "tall" or "middle-aged" are not clear-cut, but fuzzy. Indeed, it would be ridiculous to impose a threshold above which a person is "tall" and one who is a few millimetres shorter is not.

In this context, fuzzy logic tries to cope with the fact that computers cannot match the natural ability of humans to deal well with imprecise information. Fuzzy logic represents a step further in the treatment of a concept that was stressed many times throughout this research work, i.e., the heterogeneity of agents. As said, the agents in a system might belong to the same class but they might differ slightly or significantly in their characteristics. This includes the subjective way agents might perceive and respond to certain properties of the system. Consider the following example, partially inspired by Izquierdo et al. (2015). Let us imagine a system of reading recommendations for language learners. The objective of the system is to recommend to individual language learners a set of readings whose difficulty matches their level of fluency. This could be achieved by asking readers to rate the readings in terms of difficulty (for example on a scale from 1 to 10, where 1 is "extremely easy" and 10 is "extremely difficult"), along with their self-assessed level of fluency (say, from basic to advanced). Ideally, this system would be recommending readings that are accessible to readers of the same level. However, such a system (or at least, a system of this sort that is accurate and consistent) is very hard to implement. One of the reasons is that users are faced with a number of fuzzy concepts at various levels. For example, users usually have different understandings of the words "easy" and "difficult". Words like "extremely" might be interpreted as carrying different amounts of intensity. When asked to self-assess their level of fluency, users might have radically different understandings of what it means to have basic knowledge of a language or being fluent in it.^7^ Consequently, one might consider implementing a framework of shared definitions to correctly assess users' evaluations. Such a framework might have varying degrees of precision. On the one side, very general definitions are quick and easy to handle for users but might not lead to any significant improvement. On the other, a long list of very detailed descriptions could be cumbersome and discourage users. In order to find the appropriate level of detail, one might consider simulating the recommendation system in a computer environment. The model would reproduce individual users, each with their own (randomly assigned) level of fluency and understanding of the various concepts mentioned above.^8^ The agents would then be presented with readings (whose difficulty is exogenously determined) and asked to rate them selecting from a list of descriptive words, according to their interpretation of these words. The implementation of a framework of shared definitions would be represented by a lower or greater variation of these understandings among users. The objective of the model would be to determine to what extent leaving room for the concepts mentioned above to be fuzzy causes a mismatch in the recommendations and the actual level of users. This way it is possible to determine how precise the framework of shared definitions should be, in order to find an optimal compromise between a superficial set of indications and a cumbersome and tedious list of descriptions.

#### Natural language processing and machine translation

As was said many times, agent-based models are highly dependent on reliable and accurate decision rules for agents. Grounding these rules in qualitative and empirical studies has often proved a successful though labor-intensive practice, because it is based on the direct observation of human behaviour. Recently, an increasing number of authors have suggested leveraging the vast amount of text data available today to model human cognition. Padilla et al. (2019), for example, suggest using natural language processing (NLP) to analyse the description of social phenomena in order to extract potential ABM specifications from unstructured narratives. This would allow to bridge the gap between simulation experts (those who have the technical skills to develop ABMs) and domain experts (those who provide information about the phenomenon at stake in order to inform the model). Another interesting idea is the use of NLP to model the role of associations in judgment and decision-making, which represents a major challenge in the creation of realistic agents. Bhatia (2017) notes that, through associations, individuals are able to process co-occurrences and statistical regularities on the basis of their past experiences in a relatively fast and effortless way. Such evaluations, whether correct or not, play a central role in the individual decision of the behavioural response to a stimulus. He proposes using word embeddings (vector-based representations of words) to generate realistic agents.^9^ He discusses how often people fall in the so-called "conjunction fallacy", a common cognitive fallacy occurring when the joint probability of a set of conditions is erroneously believed to be higher than the probability of a single general one. This fallacy, first discussed by Tversky and Kahneman (1983), is due to the fact that a more detailed description of an event (for example, an individual having a certain profile) can deceptively seem more "representative" of the population from which it is drawn and hence more likely. Bhatia (2017) argues that an idea or a situation (such as a stimulus) can be represented as a vector of the words that make up its description in terms of a given number of dimensions. The same can be done to represent possible reactions (such as the potential behavioural responses to a stimulus). One can then calculate the distance between these vector space representations (usually as the cosine of the angle for each pair of vectors) to determine the most likely reaction to an input. Following this line of reasoning, Runck et al. (2019) argue that word embeddings are able to capture people's cognitive biases and therefore reproduce more realistic behaviours. Therefore, the authors point to the fact that informing agents in this way may help overcome the too often fallacious assumption that agents behave rationally.

In addition to its application to ABMs, the use of natural language processing is very promising in the context of policy making. Many applications of NLP to policy issues were proposed in the past few years, such as using machine translation and fixed-phrase translators in emergency settings to enable communication between medical staff and refugee patients when they cannot communicate in a common language (Spechbach et al., 2019). Many other examples can be discussed. For example, one could wonder about the role of language-related computer-based methods, such as machine translation, in the provision of multilingual services. One might hastily conclude that, in an ideal future, a sufficiently advanced machine translation system might be the key to a world free of language barriers. In the context of minority language protection, being able to provide accurate translation across various languages could be seen as a measure in support of the diffusion of less spoken languages. Besides, in this ideal context, there would be no particular pressure to acquire skills in a more spoken language, in that one could simply rely on machine translation. One might even go as far as to say that reliable machine translation would make language rights (a form of human rights specifically concerning languages and their use) obsolete. Indeed, in an ideal (admittedly, sci-fi) scenario in which one could, say, wear a device that provides highly accurate translation of spoken and written language on the spot, one could easily live one's life in one's own native language.^10^ However, the discussion would not be so simple. One could even argue that machine translation might actually work against minority languages. As a matter of fact, artificial intelligence (and, consequently, all machine-mediated services) improves significantly when it is trained with an increasing amount of information. Given that, as of today, the availability of corpora to train machine translation systems is strongly skewed towards a very limited number of languages, extremely accurate machine-based translations are very unlikely to exist for all language pairs. As a consequence, translation between some specific pairs of languages would be much more accurate than other combinations. Eventually, machine translation would simply incorporate and transpose to a virtual context the already existing bias that favours widely spoken languages. These as well as other considerations are challenges that will have to be faced by policy makers willing to exploit the potential of computer-based methods.

## Conclusions

Throughout this paper I tried to show that computational methods can play an important role in the field of language policy. This role has been largely overlooked until recently. This does not only concern simulation-based methods, but computational methods in general, such as natural language processing and other machine learning-based methods. This is largely justified by the fact that, as I have discussed at length, language matters display many traits commonly associated with complex systems. As a matter of fact, numerous scholars have observed over the past few years that language-related issues are extremely multi-faceted and that a single disciplinary perspective can only shed light on one side at a time. Only genuinely interdisciplinary approaches can hope to capture more complexity. Nevertheless, while such approaches are strongly supported by numerous scholars in the scientific community, they still represent the exception rather than the rule. Drawing from many disciplinary backgrounds, a complexity theory approach represents a step in the direction of spelling out with greater accuracy all the causal links involved in language issues.

I would like to stress once more the great potential of simulation models, as well as computational models in general, for the purposes of policy making in all its phases, from development to evaluation. The possibility to experiment in a virtual world that is fully controlled by the policy maker represents a major advantage over randomized controlled trials involving actual people. Once the policy model is developed, various scenarios can be simulated under different conditions with virtually no cost. Conversely, setting up multiple controlled trials can be very costly, not to mention the fact that it might have important ethical implications. Moreover, controlling for the impact of external variables can be really challenging in real-life, while it is an almost trivial task in a simulated environment. Furthermore, even when there is little data for calibration and validation, a simulation model can be very useful in providing an overall idea of the type of impact that one should expect from a given policy. To conclude, I shall say that the culture of computational modelling will need to spread in the policy making environment for the full potential of simulation models to be exploited.

## Data Availability

The manuscript has no associated data.

## Footnotes

1 With few exceptions, such as Guaraní, which is spoken by 6.5 million people and enjoys official status in Paraguay, Bolivia and some areas of Argentina and Brazil.

2 Directive 2010/64/EU of the European Parliament and of the Council of 20 October 2010 on the right to interpretation and translation in criminal proceedings. This directive draws on Article 14 of the International Covenant on Civil and Political Rights, which, among other things, ensures the right of an individual involved in a proceeding “[t]o have the free assistance of an interpreter if he cannot understand or speak the language used in court” (International Covenant on Civil and Political Rights, 1976, art. 14.3).

3 The expression “black swan” is attributable to the Latin poet Juvenal, who wrote: “Rara avis in terris nigroque simillima cygno” (“A rare bird on Earth, very much like a black swan”, my translation). Being common in Southwestern and Eastern Australia, black swans were presumed not to exist by the Latin scholars who coined the phrase. For more on the idea of black swans, see Taleb (2007).

4 For a different example, the fact that the General Delegation for the French language and the languages of France (“Délégation générale à la langue française et aux langues de France”, DGLFLF) prescribed the use of the word “courriel” since June 20, 2003 (Journal officiel du 20 juin 2003), never stopped people from using the words “mail” and “e-mail”. It should be noted, however, that the DGLFLF only provides lexical resources which are meant as simple terminological recommendations and not as a way force people to use certain specific words.

5 Note that a language is a complex system in that it is also located in the “edge of chaos” previously mentioned. Words have to respect precise patterns of expression, which are provided by the grammar and are generally shared and understood by all speakers. Nevertheless, they are at the very same time subject to arbitrary use by the speakers.

6 Incidentally, it is worth noting that many of these publications appear in scientific journals other than those strictly mentioning language policy or sociolinguistics in the list of topics addressed. For example, Karhunen et al. (2018) review a long list of articles on language-related issues that were published in management journals.

7 The Common European Framework of Reference for Languages (CEFRL) tries to address specifically this issue by accurately breaking down each level of fluency in terms of skills expected from language learners.

8 I will not present here the technical details of how fuzzy logic is implemented in agent-based modelling, as they can be somewhat cumbersome. For a technical explanation, see Izquierdo et al. (2015).

9 Word embeddings map words drawn from a corpus to a vector space, while conserving semantic similarity through basic operations. This property allows word embeddings to capture well human associative reasoning (a well-known example is the relation "king"−"man" + "woman" = "queen").

10 This is in no way a new idea in the sci-fi literature. In his renown "The Hitchhiker's Guide to the Galaxy" sci-fi series, British writer Douglas Adams created the "Babel fish", a small yellow fish, which can be placed in one's ear in order for one to hear any language translated immediately into one's own native language.
